# Exploration of Clinical Ethics Consultation in Uganda: A case study of Uganda Cancer Institute

**DOI:** 10.21203/rs.3.rs-3853569/v1

**Published:** 2024-01-24

**Authors:** Mayi Mayega Nanyonga, Paul Kutyabami, Olivia Kituuka, Nelson K Sewankambo

**Affiliations:** Joint Clinical Research Centre; Makerere University; Makerere University; Makerere University

**Keywords:** ethical dilemmas, clinical ethics consultation, Uganda

## Abstract

**Introduction:**

Globally, healthcare providers (HCPs), hospital administrators, patients and their caretakers are increasingly confronted with complex moral, social, cultural, ethical, and legal dilemmas during clinical care. In high-income countries (HICs), formal and informal clinical ethics support services (CESS) have been used to resolve bioethical conflicts among HCPs, patients, and their families. There is limited evidence of mechanisms used to resolve these issues as well as experiences and perspectives of the stakeholders that utilize them in most African countries including Uganda.

**Methodology:**

This qualitative study utilized in-depth-interviews (IDIs) and focus group discussions (FGDs) to collect data from Uganda Cancer Institute (UCI) staff, patients, and caretakers, who were purposively selected. Data was analyzed deductively and inductively yielding themes and sub-themes that were used to develop a codebook.

**Results:**

There was no formal committee nor mechanism utilized to resolve ethical dilemmas at the UCI. The study uncovered six fora where ethical dilemmas were addressed: individual consultations, tumor board meetings, morbidity and mortality meetings, core management meetings, rewards and sanctions committee meetings, and clinical departmental meetings. Participants expressed apprehension regarding the efficacy of these fora due to their non-ethics related agendas as well as members lacking training in medical ethics and the necessary experience to effectively resolve ethical dilemmas.

**Conclusion:**

The fora employed at the UCI to address ethical dilemmas were implicit, involving decisions made through various structures without the guidance of personnel well-versed in medical or clinical ethics. There was a strong recommendation from participants to establish a multidisciplinary clinical ethics committee comprising members who are trained, skilled, and experienced in medical and clinical ethics.

## Introduction

Globally, health care providers (HCPs), hospital administrators, patients and their caretakers are increasingly confronted with complex moral, ethical and legal dilemmas during clinical care that call for mechanisms of resolution. Ethical issues in healthcare have moved beyond the HCP’s professional competence to salient individual, cultural, economic, political, religious, and social challenges. These include but are not limited to vagueness in informed consent and surrogate decision making, conflict in interpersonal relationships, rationing of limited resources, medical futility in intensive care units, diverse cultural interpretations of treatment, truth-telling, end-of-life decisions, and refusal of treatment. These dilemmas have led to moral distress, burn out, defensive medicine, HCPs’ dissatisfaction on the job, unsatisfactory services to patients and the reputational damage to HCPs and institutions. (1)

At times medical practice is associated with conflicting preferences, even in the HCP’s desire to act in the patient’s best interests. Such tensions and uncertainties can be due to competing values, interests, rights and abstract principles, emotional reactions and varying opinions of an unfair processes in handling clinical decisions. (2) There has been exponential growth in the application of ethical and moral judgments in addressing medico-ethical issues at the hospital bedside. The growth of bioethics in the 1960s illustrates the development of ethical decision making, for example in making choices of who should live or die in the context of scarce life support treatments as witnessed in the God’s committee. (3) To date, similar questions continue to occupy the minds of governments, bioethicists, clinical ethicists, health care workers, patients, and their families. This was very clear during the 2021 Ebola virus disease outbreak in Uganda and the 2020 COVID-19 pandemic rationing of health resources and priority setting. (4, 5)

Decision making and allocation frameworks have been proposed by a number of scholars and by WHO. (6, 7) In high-income countries (HICs), formal and informal clinical ethics support services (CESS) have been used to resolve bioethical conflicts among HCPs, patients, and their families. Well established CESS mechanisms include clinical ethics committees (CECs) and forums for moral deliberations such as ethics reflection groups and ethics rounds. (8, 9) Additionally, the characteristics, functionalities and effectiveness of these mechanisms have been documented. (10-12) However, African settings are faced with ethical dilemmas, with scanty evidence of approaches for their resolution. Limitations to formation and access to CESS may range from inadequate knowledge of these mechanisms and processes, misconceptions about ethical consultations by patients and HCPs, power imbalances between HCPs and patients or their families, lack of time, inadequate qualified ethicists as well as limited resources to establish formal ethics consultations. (13, 14)

In Uganda, patient care grapples with resource scarcity, disease burden, health-seeking issues, treatment adherence, emotional and psychosocial factors, communication gaps, limited knowledge, and slow medical technology progress. Notably, challenges for cancer patients such as transition to end of life and honoring patient choices are complex and come with moral dilemmas for healthcare providers, patients, and their caregivers in addition to causing distress among HCPs. (15) There's an urgent need for a situation analysis of clinical ethics consultation services at the Uganda Cancer Institute (UCI). Understanding the current clinical ethics service offerings is crucial for improvement and development of clinical ethics support services.

## Methods

To address this knowledge gap at the UCI, we undertook an exploratory qualitative study that employed in-depth interviews (IDIs) and Focus Group Discussions (FGD) data collection methods.

### Study setting

Established in 1965 by Makerere University in Uganda and the National Cancer Institute (NCI) in the USA, the Uganda Cancer Institute (UCI) is a renowned East African centre of excellence in oncology. (16) The UCI also plays a crucial role as a research and training facility. Handed over to the Uganda Ministry of Health, UCI now serves as a pivotal hub for cancer care. With a bed capacity of 80, it attends to approximately 200 outpatients daily, including those from Uganda, the Democratic Republic of Congo, South Sudan, and neighbouring regions. (17) UCI provides comprehensive oncology clinical care, encompassing paediatrics, gynaecology, radiotherapy, surgery, and pharmacy services. Integrated within these disciplines are palliative care, counselling, and social support services. Despite its crucial role, UCI faces understaffing, with a doctor-patient and nurse-patient ratio of 1:100 and 1:50 respectively. (18)

With a nearly 60-year history, UCI's autonomy and it being the only comprehensive cancer care facility in Uganda made it an ideal site for this study.

### Study participants

Participants, including UCI staff, patients, and their caretakers (aged ≥ 18), present at the UCI during the study, were purposively selected. Eligible individuals spoke English or Luganda, had previously faced challenging issues in their care requiring ethical resolution, and were willing to undergo audio-recorded interviews. To identify potential participants meeting the inclusion criteria, UCI social workers and counselors were contacted, and a compiled list of these individuals was then created. Only participants who provided written informed consent were recruited into the study.

### Data Collection

Data was collected qualitatively using IDIs and FGDs in December 2022. Interview questions were guided by a structured interview guide that was developed by the authors. Interviews, conducted with the participants' informed consent, were moderated by one researcher and a research assistant who took notes.

In total, 21 IDIs were conducted; 12 with UCI Staff (5 female and 7 male) and 9 with patients (5 female and 4 male). 3 FDGs were conducted; 2 with patients and 1 with caretakers. Each patient FGD consisted of 6 participants. One patient FGD had female participants only while the second had male patients only. The caretaker FGD was conducted with 10 caretakers of mixed gender, 6 female and 4 males. All IDIs with UCI staff participants were conducted in English. For patient participants, 5 IDIs were conducted in English while 4 were conducted in Luganda. One FGD with patients was conducted in English and the second conducted in Luganda The FGD with caretakers was conducted in Luganda. Luganda was a preferred language for translation because it is a commonly understood local language.

Interviews lasted between 45 minutes to 1 hour, were recorded using a digital recorder and thereafter transcribed and anonymized. Each day, Luganda audio files were translated to English and transcribed. Data collection was guided by the principle of saturation, with no new themes coming up during the interviews. (19, 20)

Two interview guides, one for HCPs and another for patients and their caretakers, were created and tested with 2 HCPs, 2 patients, and 1 caretaker to ensure the guides’ validity and reliability. The final guides were then refined to address any repetition or incomplete information. The updated interview guides were used to investigate the mechanisms employed in resolving ethical dilemmas at the UCI, factors that influenced these consultations and perspectives and experiences of stakeholders that utilized these services. Initially, the questions were formulated in English and subsequently translated into Luganda by a certified translator at the Makerere University, Department of African Languages. The Luganda translations were reverse translated into English to verify the accuracy of the translated versions.

### Data analysis

Audio recorded interviews were transcribed verbatim. The transcriptions were then prepared and entered into Nvivo 12 qualitative scientific software for analysis. The COREQ checklist was applied to ensure adherence to criteria for qualitative research. (21) The data underwent thematic analysis, and both inductive and deductive approaches to qualitative data coding and analysis were employed. With reference to the interview guide and objectives, deductive analysis involved the development of a predefined coding framework. In contrast, inductive analysis included the creation of additional codes during the transcript review which expanded the codebook. Thorough examination of the transcripts and field notes aimed at deriving meaning and interpretation from the rich data were carried out. Any pertinent aspects not initially covered in the codebook prompted the generation of new codes to address new areas of inquiry.

For purposes of validating and verifying findings, data was triangulated through taking notes with a research assistant that was experienced in qualitative research. Where any inconsistencies in data sets during transcription were identified, participants were contacted to clarify what they meant. We also performed member checking on 4 participants. Transcripts for these 4 participants were shared with them for review and confirmation on accuracy of information transcribed.

### Ethical Considerations

The head of research, counsellors, social workers, patient advocacy groups at the UCI were consulted to identify potential participants. The purposively selected participants were contacted via email, phone/SMS to inform them about the study, its design, and potential risks and benefits after which they were asked to provide written informed consent. Additionally, interviews were scheduled and conducted in a quiet space conducive to privacy when conducting the FGDs and IDIs.

Participant information was kept confidential with identifying information (such as name)encrypted and stored separately from any study data. Only the researcher conducting interviews and the research assistant had access to the password protected dataset. Informed consent forms, recruitment materials and interview notes were stored in lockable cabins, under lock and key, with only restricted access to the research team.

This was a minimal risk study whose inconvenience to participants were some discomforting sensitive information, sociodemographic, clinical, and behavioral questions. Participants were free not to take part or to discontinue participation in the study at any time. Potential participants were informed that refusal to participate or withdrawal from the study would not affect a patient’s treatment plan nor affect the staff’s employment (in the case of HCPs) at UCI. The participant’s decision to participate or not was kept confidential.

## Results

All UCI staff participants had at least attained tertiary education, with the highest level of education being post-doctorate level. However, no training specifically focused on bioethics or clinical ethics.

Based on the analysis, three main themes and five sub-themes were identified: the first as mechanisms for resolution of ethical dilemmas with sub-themes as ethical issues, ethical dilemmas, and existing measures/policies to guide resolution of ethical dilemmas. The second main theme was factors influencing clinical ethics consultation. The third was perspectives and experiences of healthcare workers, patients and caretakers of the clinical ethics consultation at the UCI and had subthemes of recommendations for improved clinical ethics consultation and considerations for establishing a clinic ethics committee.

### Main theme 1: Mechanisms for resolution of ethical dilemmas

Many patients and caretakers did not know of any existing mechanisms utilized to resolve ethical dilemmas nor where to report when faced with ethical dilemmas or issues.

Honestly, for the time I have spent in the hospital, I am not aware of any mechanism put in place to address such issues. I do not think there are formal systems or structures for solving dilemmas.

(FGD 3, respondent 4)

The absence of a formally established committee, such as a **clinical ethics committee**, to provide guidance on resolving ethical issues and dilemmas was notable. Instead, such concerns were informally reported and addressed with varying significance and approaches. According to UCI staff, six meeting forums were employed for resolving ethical dilemmas, employing a top-down approach where healthcare workers initiated all discussions.

#### Individual consultation

Some patients and their caretaker consulted with counsellors, social workers, or their doctors, who helped them resolve ethical issues at individual level.

“Me, I'm an open person, I always go to the senior doctor and tell him what is hurting me. The senior doctor always helps me to get a solution. But many patients here fear to speak up because they fear they will not be treated. Me I speak my mind.”

(IDI-07)

#### Tumor Board meetings

Some ethical conundrums were discussed in tumor board meetings where complex cancer cases were held.

“We use the tumor boards. These tumor boards involve many disciplines; medical oncologists, radiation oncologists, nurses, pharmacists, radiologists, pathologists. So there when patients are discussed, an appropriate treatment plan is decided on by the team. And in the department, like in radiotherapy, we have Thursday departmental meetings, where we discuss patients before they start treatment.”

(IDI-18)

#### Morbidity and mortality meetings

Some ethical issues were reported to be discussed during morbidity and mortality meetings. With an aim of improving service delivery, any social and ethical issues that might have impacted treatment plans were addressed in this forum with strategies to mitigate them from recurring.

“…what could have caused the death of the patient? was the death avoidable? is the cause attributed to negligence. Such issues can still be addressed by the morbidity and mortality manager at morbidity and mortality meetings…”

(IDI-10)

#### Rewards and Sanctions committee meeting

Previously known as the Disciplinary Committee, the rewards and sanctions committee were believed to handle some ethical issues and dilemmas as part of the disciplinary inquiries.

“The Rewards and Sanctions Committee is composed of 5 people. It used to be called the disciplinary committee in the old days. We thought it would be good to motivate the staff that performed well at work, so we changed the name. Individuals with complaints forward them to that committee and then the committee goes through them and decides what to do.”

(IDI-18)

**Clinical departmental meetings** were also utilized by different HCPs to resolve address ethical conundrums as challenges faced by different clinical departments staff.

“Team meetings, or departmental team meeting handle such scenarios and decide what to do…”

(IDI-15)

**Core management meetings** were weekly leadership meetings that were also reported to have been an opportunity for different UCI staff to share any challenges of complex decision making during clinical care.

“….Issues can also be addressed by the UCI core management. So whatever issue that comes up, depending on the level of its magnitude, it can be addressed by whatever level of managers that we have.”

(IDI-10)

Subtheme 1: Ethical issues encountered by healthcare providers, patients, and caretakers

Ethical issues that were reported to be resolved by the mechanisms explored included paternalism, informed consent, privacy, and confidentiality.

Paternalism: A considerable number of patients and their caregivers held the belief that their physicians possessed comprehensive knowledge and depended on their expertise and experience to provide sufficient care.

“The doctor knows better and has experience about the treatment I am receiving so I cannot object to what he decides. Even if I am feeling so weak and the doctor says I have to continue with the chemotherapy, I continue because I am not the doctor…”

(IDI-07)

Privacy and Confidentiality: On observation, the UCI had a significant number of patients but limited space available for triage during examination. The high patient volume made it impractical to assess individuals in an environment that guaranteed privacy. One participant reported discomfort during discussion of her case at one of the expert forums.

“They took me to tumor board to discuss my breast cancer issue. They made me remove my blouse and expose my breast as the team discussed about it. I felt so uncomfortable, but I had nothing to do. I need help. All I want is to be fine.” Started crying…

(FGD-02 – Respondent 4)

Subtheme 2: Ethical dilemmas encountered by healthcare providers, patients, and caretakers

Staff, patients, and caregivers at the UCI encountered challenging scenarios where decision-making appeared complex. Challenges included conflicting beliefs and values influenced by religion, culture, and interpersonal relationships, issues related to benevolent deception, treatment choices based on financial considerations, power imbalances, healthcare resource rationing, and conflicts of interest.

Conflicting beliefs and values: Numerous patients held religious and cultural beliefs that diverged from the conventional cancer treatment options endorsed by doctors, leading to complexities in decision-making. Beyond socioeconomic factors, decisions regarding care were sometimes influenced by the perspectives of friends, family, and other patients within the cancer community. Some individuals mentioned concurrently using traditional herbal remedies alongside chemotherapy, posing a challenge for healthcare providers in selecting appropriate treatment options. Healthcare professionals reported concerns that herbal medicines could potentially interact with chemotherapy, adversely affecting their patients’ prognosis.

“… my friends and family told me to try drinking herbal medicine and I am using them also. They gave me the number for the herbalist. I also know cancer patients can’t be healed; you just die. So, I don’t know what to do…”

(FGD-02 – Respondent 2)

Autonomy – informed consent: Healthcare providers (HCPs) faced challenges in determining the appropriate course of action for minors whose parents prioritized religious and cultural beliefs. It was noted that a lack of cooperation from parents added complexity to decision-making, even when HCPs aimed to act in the best interest of the minors. Issues were raised concerning competent minors with no legal capacity to consent. Many physicians were reluctant to provide treatment to these children due to concerns about potential legal repercussions that could adversely affect both their personal reputation and that of the hospital. They also expressed reservations about the lengthy legal processes in Uganda, which were deemed time-consuming and had the potential to interfere with their daily duties of treating patients.

While healthcare providers are bound by the Hippocratic oath, which obligates them to minimize harm and maximize the benefit to patients during clinical care, it was reported that some patient preferences conflicted with the physician’s duty to provide care and enhance their quality of life, especially for empathetic providers.

“A child of 16 years comes in alone to get their chemotherapy, but they do not get it. You know why? Because she is a minor with no caretaker to consent, yet they need chemotherapy. This chemotherapy comes with side effects, and these children need support from caretakers or guardians. What if this child dies, who is responsible? What if they ask who consented on their behalf? Tell me, what would you do if you were the doctor? It becomes very difficult to decide how to help this child.”

(IDI-08)

Resource allocation: The allocation of scarce resources during rationing posed ethical challenges at the UCI. Limited resources, including human resources, technology, and supplies, were reported. During the data collection period, floor cases were observed on the wards. Many caretakers reported inadequacies in wheelchairs, leading them to carry their patients to observation rooms. A significant number of patients conveyed that their numbers did not allow all of them to receive timely radiotherapy, leading to disease progression and a bleak prognosis. Compounding this issue, the occasional dysfunction of radiotherapy machines meant that some patients missed their scheduled radioactive treatments, a situation also reported for patients on the surgery list. With specific days allocated for different surgeries, many patients had lost hope of their turn ever arriving.

Nurses highlighted challenges with the insufficient availability of oxygen ports, creating dilemmas about prioritizing patients in need of oxygen. They also mentioned a shortage of nursing staff relative to the high number of patients, making it impossible to attend to everyone efficiently and equally.

“Now, I want you to imagine, you are one nurse or two working on forty patients who are critically ill. Remember, one patient alone can make you extremely tired, but now you’re having forty critically ill and don’t what to lose any life. You get confused about whom to start with. By the time you complete, you don’t want anybody talking to you, you are really tired and burnt out. Of course, they will say you are ignoring them but still there is no way you can run in between and suspect that may be this one wants attention, you won’t even know how somethings happen…”

(IDI-05)

Truth telling: Some caregivers expressed a preference for keeping their patients unaware of their cancer diagnosis, urging healthcare providers to administer treatment without disclosing the nature of the patient's condition. Conversely, others were comfortable with their patients being aware of the cancer diagnosis but requested doctors to refrain from sharing all details. Many physicians observed that these situations presented challenges to their duty of veracity, or truthfulness, to their patients.

“I do not want my patient to know everything. Sometimes when I go to the doctor's, I use English because the patient does not understand English. Ha ha ha… I don't want my patient to lose hope because he is always thinking about death and says he is ready to die. I do this to help him. Imagine if he hears that some organ has been affected by chemo, I would be the one to suffer. I need him to receive his treatment in peace.”

(IDI-13)

Subtheme 3: Existing policies and measures to guide resolution of ethical dilemmas

The Uganda Cancer Institute lacked established policies specifically addressing clinical ethics consultations. Reports indicated that the development of an ethics code of conduct at UCI was underway, which would supplement the existing client charter and professional codes of conduct. These documents collectively aimed to provide guidance for healthcare providers in their decision-making processes.

“…At the moment, there is a document which is going to come out in the next one or two months about ethical code of conduct for UCI, and I'm spearheading it, it's almost in its terminal stages…”

(IDI-18)

“No, we don’t have any specific ethical documented guidelines. Currently, we base on what is clinically regarded as right or wrong.”

(IDI-19)

### Main Theme 2: Factors influencing clinical ethics consultations at the Uganda Cancer Institute

The effective resolution of ethical dilemmas at the Uganda Cancer Institute (UCI) was reportedly influenced by several factors. These included a lack of sufficient space to ensure privacy, limited knowledge in medical ethics, and time constraints for UCI staff. The absence of clear mechanisms for resolving ethical dilemmas, coupled with power imbalances, was identified as a significant barrier to seeking resolution. Certain UCI staff members also highlighted the absence of institutional policies and guidelines on ethical dilemma resolution as a substantial impediment to providing adequate ethics consultation services. Additionally, a lack of resources to compensate healthcare providers specifically dedicated to addressing ethical dilemmas was raised by several participants.

Many patients and caretakers reported a lack of awareness regarding where to seek guidance for the resolution of the ethical dilemmas they faced. Moreover, these participants were willing to utilize existing platforms or committees if they knew where to find them.

“The workload! These doctors are overwhelmed by the patient numbers. They see so many patients. Some of them must handle administrative and human resource issues too. So, I don’t think such people can concentrate and come up with a good structure or find a vibrant committee that they can come to or reach out to a common man in terms of emphasizing what to be done here and there. I don’t think that time is there. They may contribute to your idea but will not come by to discuss individual ethical dilemmas.”

(IDI-04)

“The challenge, aah… I tried to talk to someone, but as I said, they are some people that are like untouchable, some people are aware that there is nowhere you can report them, may be to God. You see something, but someone is like an elephant so just keep quiet and suffer mentally about it.”

(IDI-03)

#### Main theme 3: Experiences and perspectives of patients and caretakers following utilization of existing mechanisms for resolution of ethical dilemmas

Concerns were raised among UCI staff regarding the ethical competence of members comprising various committees. Some HCPs, patients, and caretakers expressed doubts about the suitability of existing forums to address clinical ethics dilemmas and questioned their overall effectiveness.

“…even if some ethical issues might arise during these meetings, there is no time to discuss these issues. The agendas for the meetings are even so different and ethical issues are not priority. Take an example, tumor board meetings are for discussing complicated cases in terms of disease not ethics. The time for tumor board is also about 2 hours and they can discuss one patient for like 35 minutes. Now, if the time is not even enough to discuss all proposed patients, where will the time to discuss ethical issues come from? These doctors do not have time, they have to go and see patients.”

(IDI-15)

Subtheme 1: Recommendations for improved clinical ethics consultation

Most study participants overwhelmingly advocated for the establishment of a dedicated multidisciplinary clinical ethics committee, trained in clinical ethics to handle ethical dilemmas. Their reservations however primarily revolved around concerns related to limited funding and the absence of policies to support the establishment of such a platform.

“We need a clinic ethics committee to oversee all the clinical ethics aspects that are going on at UCI…. we need it definitely. That is one thing that is missing at UCI.”

(IDI-03)

Subtheme 2: Considerations for establishing a clinical ethics committee

Participants outlined the composition of the envisioned clinical ethics committee and the qualities deemed essential for its members. A frequently mentioned preference was for a full-time, diverse committee comprising healthcare professionals, expert patients, clergy, and lay individuals. Additionally, participants emphasized the committee's responsibility to formulate guidelines and policies for addressing ethical issues and dilemmas.

Furthermore, participants highlighted the importance of committee members possessing knowledge and training in medical and clinical ethics, along with a combination of soft and technical skills to effectively engage with people in a considerate and proficient manner.

“I think a full representation would be good because at the different service points, different people face different ethical issues. Team radiotherapy, team nuclear medicine, the pharmacist, the doctor as well as having survivors or patients come on board.

(IDI-10)

“Someone's behavior is important. Like someone should not be short tempered. One should be calm and able to handle different people without bias or favoritism.”

(FGD-02, Respondent 4)

## Discussion

There was no formal forum or mechanism utilized to resolve ethical dilemmas at the UCI. The study identified six fora that patients, caretakers and UCI staff utilized to resolve ethical issues and dilemmas. Patients and caretakers addressed straight forward ethical issues through one-on-one consultations with their healthcare providers. In contrast, complex ethical dilemmas were typically deliberated on in formal settings such as tumor board meetings, morbidity and mortality meetings, rewards and sanctions committee meetings, core management meetings and clinical departmental meetings, all of which followed scheduled roosters. These approaches are similar to clinical ethics resolutions used in different settings globally. (22, 23) Unlike the structured clinical ethics consultation services prevalent in developed nations, fora utilized to resolve ethical dilemmas at the UCI, while integral to their established roles, have been scrutinized for their implicit and non-ethics-focused nature. While these existing platforms serve their intended purposes, they fall short in adequately resolving complex ethical issues. An intriguing observation also emerged as healthcare providers found themselves juggling multiple roles across different meeting platforms, leaving them with insufficient dedicated time to tackle clinical ethics comprehensively.

### Individual level consultations

Resolution of ethical issues was advanced through intuition, education, and work experience of the different HCPs. The patients and caretakers’ motivations to report issues included trust, nature of the problem, education level, and previous experience with the HCP. There is wide consensus in the use of intuition in clinical practice and moral judgements to resolve ethical issues and dilemmas. (24, 25) Proponents of this argue that it reduces turn around time in resolving dilemmas, increases a ‘personal touch’ and promotes flexibility and shared decision making. Intuitive clinical judgements, have however, reinforced the traditional authority of the doctor tending to paternalistic health care. This ignores shared decision making which can negatively impact on quality of care as the patient’s values and beliefs are undermined. It further limits autonomy and empowerment of patients especially those with low literacy levels that cannot ably speak for themselves. (26) In addition, paternalism does not allow for exploration of dynamic and challenging ethical issues that arise during clinical care. HCPs who are accustomed to deciding for their patients are likely to be challenged with ethical quandaries when they interact with more assertive and ethnocentric patients.

Some doctors at the UCI demonstrated a reliance on experience as a significant factor in resolving both medical and ethical issues. They reported a preference for utilizing previously employed approaches to address similar cases in the future. However, this reliance on experience may pose challenges, as it assumes that the context of similar cases remains constant. In reality one might encounter conflicting situations that demand a different course of action. Evidence supports the idea that individuals with extensive experience tend to easily recognize issues they are familiar with, applying casuistry and reasoning strategies to navigate them effectively. (27)

Contrastingly, a study conducted among health science students at the University of Southwest New Mexico revealed a different perspective. The findings suggested that as students gained more experience, their ability to make ethical decisions actually decreased. (28) This discrepancy highlights the complexity of the relationship between experience and ethical decision-making, indicating that assumptions about the consistency of similar cases may not always hold true.

### Tumor board meetings

During these meetings, respondents highlighted that a multi-disciplinary team of experts convened weekly to scrutinize intricate cancer cases. The goal was to reach a consensus on diagnosis and treatment plans, all aimed at optimizing patient care. This collaborative approach mirrors practices in Rwanda, Kenya, and Botswana, where similar strategies are employed in cancer settings to address complex dilemmas within cancer wards. (23, 29-31) Intriguingly, only a minimal number of respondents in this study experienced this collaborative approach. Notably, the tumor board meetings were not primarily convened for ethical and moral case deliberations. However, these considerations naturally surfaced as integral components of the comprehensive discussions centered around the holistic care of the patient.

### Mortality and Morbidity Meetings (M&M)

The objective was to enhance patient safety and the quality of care through a monthly review of introspective cases involving patient deaths, including their causes and treatment outcomes. They focused on identifying ways to improve care and prevent similar errors in future analogous cases. In the United States, regular M&M meetings are mandatory for hospitals as part of their accreditation and maintenance process.(32) A study conducted by D.L. Clarke et al demonstrated how these meetings yielded evidence of errors and their potential causes within the trauma care staff. This, in turn, contributed to the prevention of surgical errors and the overall improvement of patient care in the South African setting. (33)

### Rewards and sanctions committee meetings

Traditionally, the prevailing approach to human resource management involved disciplinary proceedings as a means of penalizing personnel for professional misconduct and inappropriate conduct. In Uganda, this practice is codified in the public standing orders and the Patients Charter. (34, 35) A recent initiative at UCI, spearheaded by departmental heads, is the rewards and sanction committee, designed to commend commendable conduct and penalize unacceptable professional practices. Specifically, the committee addresses violations of professional ethics and instances of malpractice reported by patients and caregivers. The aim is to ensure justice for patients and encourage them to report such cases. However, a lingering question is whether a rewards and sanctions committee is equipped to adequately address the more nuanced moral and ethical dilemmas and how to establish the criteria for determining what qualifies as an ethical issue or professional misconduct.

### Core management meetings

The delivery of high-quality healthcare involves a concerted and teamwork-oriented approach, engaging both clinical and administrative staff. Management meetings are pivotal in directing the focus towards hospital performance, patient health, quality of care, and efficiency outcomes. These gatherings serve as a platform for creating a conducive practice environment, supporting executive management, resolving conflicts, solving problems, fostering team cohesion, and facilitating continuous professional development. Fundamentally, these meetings, orchestrated by the governing body, are geared towards enhancing both management and clinical outcomes. (36-38) Through collective thinking and contributions, innovative solutions to clinical challenges are generated, leading to the refinement of hospital administration. An illustrative study highlighted how contributions from all clinic doctors in such meetings resulted in an enhanced understanding of the problem and a shared sense of well-being. (39)

### Clinical department meetings

At the UCI, these meetings serve as forums for presenting patient-related matters, encompassing ethical considerations, patient welfare, clinical narratives, teambuilding and initiatives aimed at enhancing patient quality. In a South African study, nursing unit managers allocated 25.8% of their time to direct patient care, which involved addressing patient issues (37).

## Establishing Clinic Ethics Committees

Many developed countries, including the USA, Norway, Singapore, Canada, Germany, Netherlands, and Slovakia, have not only legally mandated the formation of clinical ethics committees in every hospital but have also proactively aligned their visions and goals with institutional objectives, such as enhancing patient care and satisfaction. (40-44) These committees have proven effective in resource allocation, cost reduction, improved quality of care, and alleviating moral distress among healthcare workers. (12, 45-49)

Curiously, despite its 60-year history, the UCI lacks such a committee, prompting questions about the reasons for this absence. Unlike the countries in the western world, the UCI lacked policies to support clinical ethics support services such as a clinical ethics committee, making their prioritization difficult. There is also no evidence of support for such services in Uganda at national and hospital level, yet this support is vital for the prioritization of the existence and functionality of clinical ethics committees through allocation of budgets for these programs and ensuring protected time for individuals that provide clinical ethics services.

In developed countries, individuals providing clinical ethics support services are also trained and experienced in ethics, have sufficient knowledge, skills and character traits to address the range of ethical challenges brought to them. (50, 51) In fact, standards for assessment of core competencies and skills for clinical ethics consultation have been developed for efficiency in operations and easy pooling of experts for consultation. (52, 53) In the United States, formal apprenticeship training programs that qualify one to be a clinical ethics consultant have also been developed (54, 55) Clinical ethics training in Uganda, however, is not as abundantly available as that of the first world countries. Although basic knowledge of ethical principles is taught in medical, nursing and pharmacy schools, it is not sufficient to meet clinical ethics challenges in the real world. (56, 57)

Plans to elevate the UCI into a leading centre of oncology service delivery, training, and research in East and Central Africa are underway. The institute is already experiencing a surge in patients from various regions across Uganda and neighbouring countries, significant infrastructure investments, introduction of advanced oncology services, and increased involvement in sophisticated research. The inevitably growing complexity of clinical ethics issues at UCI necessitates robust clinical ethics consultation services. The study revealed the potential bottlenecks that need to be addressed including the high cost of setting up such a service; the need for training of Clinical Ethics Support Service providers in clinical ethics; and the existence of multiple committees with overlapping responsibilities towards resolution of ethical dilemma.

## Study limitations

The subjectivity of responses from qualitative questions make it impractical to generalize the findings of the study to all hospitals in Uganda. Further explorative studies in different regions of the country are needed to understand mechanisms they utilize to resolve ethical dilemmas and recommendations of what approaches would be feasible in their context. This cross-sectional study was also limited in accounting for the entire continuum of clinical ethics consultation since data was collected at a specific point in time. Despite these limitations, this study demonstrates the need for establishment of clinical ethics support services in different hospitals in Uganda.

## Conclusion

This qualitative explorative study conducted at the UCI revealed six mechanisms for resolution of ethical dilemmas. HCPs as individuals or teams at existing non-ethics related meeting forums attempted to resolve cases that involved ethical quandaries without any solid ethical evidence for making thoroughly thought-through decisions. These approaches were implicit, with stakeholder uncertainties about their effectiveness in resolution of ethical dilemmas.

There is need to establish a policy guided multidisciplinary clinical ethics committee at UCI with initial and continuous training of its members in clinical ethics.
